# Perspective Biological Markers for Autism Spectrum Disorders: Advantages of the Use of Receiver Operating Characteristic Curves in Evaluating Marker Sensitivity and Specificity

**DOI:** 10.1155/2015/329607

**Published:** 2015-11-08

**Authors:** Provvidenza M. Abruzzo, Alessandro Ghezzo, Alessandra Bolotta, Carla Ferreri, Renato Minguzzi, Arianna Vignini, Paola Visconti, Marina Marini

**Affiliations:** ^1^Department of Experimental, Diagnostic and Specialty Medicine, School of Medicine, University of Bologna, Via Belmeloro 8, 40126 Bologna, Italy; ^2^Don Carlo Gnocchi Foundation ONLUS, IRCCS “S. Maria Nascente”, Via Alfonso Capecelatro 66, 20148 Milan, Italy; ^3^ISOF, CNR, 40129 Bologna, Italy; ^4^Comune di Bologna, 40129 Bologna, Italy; ^5^Section of Biochemistry, Biology and Physics, Department of Clinical Sciences, Faculty of Medicine, Polytechnic University of Marche, 60128 Ancona, Italy; ^6^Child Neurology and Psychiatry Unit, IRCCS Institute of Neurological Sciences, 40139 Bologna, Italy

## Abstract

Autism Spectrum Disorders (ASD) are a heterogeneous group of neurodevelopmental disorders. Recognized causes of ASD include genetic factors, metabolic diseases, toxic and environmental factors, and a combination of these. Available tests fail to recognize genetic abnormalities in about 70% of ASD children, where diagnosis is solely based on behavioral signs and symptoms, which are difficult to evaluate in very young children. Although it is advisable that specific psychotherapeutic and pedagogic interventions are initiated as early as possible, early diagnosis is hampered by the lack of nongenetic specific biological markers. In the past ten years, the scientific literature has reported dozens of neurophysiological and biochemical alterations in ASD children; however no real biomarker has emerged. Such literature is here reviewed in the light of Receiver Operating Characteristic (ROC) analysis, a very valuable statistical tool, which evaluates the sensitivity and the specificity of biomarkers to be used in diagnostic decision making. We also apply ROC analysis to some of our previously published data and discuss the increased diagnostic value of combining more variables in one ROC curve analysis. We also discuss the use of biomarkers as a tool for advancing our understanding of nonsyndromic ASD.

## 1. Definition of ASD

The Diagnostic and Statistical Manual of Mental Disorders, fifth edition (DSM-V), issued in May 2013 by the American Psychiatric Association, provides new diagnostic criteria for Autism Spectrum Disorders (ASD), which now includes Asperger syndrome, classic autism, childhood disintegrative disorder, and pervasive developmental disorders not otherwise specified. It classifies ASD by levels 1 to 3 for mild, moderate, or severe, based on the degree of support the patient requires.

Occurrence of ASD is four to five times more prevalent in males than in females (1 in 42 boys versus 1 in 189 girls); the Centers for Disease Control and Prevention (CDC) estimated in 2014 that 1 in 68 children aged 8 years was affected by ASD in USA [1]. Another recent estimate [2] put the burden of ASD on 1 out of 132 persons (i.e., 7.6 per 1000 persons), with little variation around the world. This discrepancy may reflect both a real increase in the occurrence of ASD and in its diagnosis (in 2012 CDC estimated the rate of ASD in US children to be 1 out of 88) and the fact that ASD diagnosis is often “lost” when children progress into adulthood, being replaced by a “generic” intellectual disability and/or hidden under late-developing neuropsychiatric illnesses [3].

Affected children usually suffer from impaired social interactions, speech disabilities—ranging from language delay to lack of speech, repetitive and/or compulsive behaviors and echolalia, hyperactivity, deficits in memory, learning, motor skills, or other neurological functions, abnormal excitability, and hyper- or hyposensitivity to sensory stimuli, anxiety, and difficulty to adapt to new environments/habits. Frequent association with comorbidities such as sleep and gastrointestinal problems has been also reported [4, 5]. A recent review [6] points out the four broad domains of development that are predictive of ASD: sensory-motor, attentional, social-emotional, and communication. Deficits in these areas may appear as early as 6–9 months of age, although most manifest during the second year. Reliable diagnosis can be made by an experienced physician around age 2; however many children do not receive a final diagnosis until much older [7, 8].

## 2. Etiology: Genetics, Environment, and Their Relationships

Betancur [9] listed 103 disease genes and 44 genomic loci recognized in ASD subjects. Among them, 99 genes were classified as* syndromic autism genes*, since the autism trait arises within the context of a complex syndrome with known genetic origin, such as the fragile X, the tuberous sclerosis, or the Rett syndrome. These single gene disorders account for 3–5% of ASD.

Moreover, advances in genetic testing, notably chromosomal microarray analysis, enabled the identification of de novo Copy Number Variations (CNV) in about 30% of affected children. In this way, about 300 rare ASD-associated CNV regions have been identified [10, 11]. Besides CNV, more than 500 single gene mutations have been identified by whole-exome and whole-genome sequencing [12, 13].

Several lines of evidence support the notion that genetics may play some role also in the remaining 70% of ASD cases. One of the more convincing of these evidences is the high hereditability of ASD. A very high concordance among homozygote twins (over 90%) was recorded in a 1995 study [14]. The genetic complexity of ASD is however supported by the low linkage association in siblings as well as in dizygotic twins, which have only a 6% concordance [14]. Moreover, a study examining parents of 69 people with ASD and parents of 52 controls showed that parents of ASD subjects presented mild forms of autistic-like features [15], the same “broader autism phenotype” recognizable in ASD siblings. Based on these observations, it is reasonable to conceive nonsyndromic ASD as a complex genetic trait, resulting from the combination of multiple de novo mutations, CNV, and rare genetic variants, with possible additive effects, which may account for the high heterogeneity in clinical presentation. In a recent review [16] Bourgeron compared all available information on the genetics of early-onset neurodevelopmental disorders in order to identify a common core of altered pathways affecting neuronal homeostasis. Pathways associated with early-onset neurodevelopmental disorders fall in the domains of cytoskeletal organization, synapse, translation, chromatin remodeling, and metabolism. However, despite similarities and overlapping of symptoms common to most if not all early-onset neurodevelopmental disorders, in particular the presence of epilepsy and of cognitive impairment in many ASD patients, and despite the marked heterogeneity of clinical presentation of ASD, specific clinical traits characterize ASD and lead to specific diagnosis. Hence, the quest for the identification of biomarkers is able to focus on the core ASD symptoms.

Alongside with genetic factors, the concurrence of a multiplicity of environmental factors is strongly emerging in the etiology of ASD. In contrast with [14], a more recent study, which examined 192 pairs of twins [17], concluded that “susceptibility to ASD has moderate genetic heritability and a substantial shared twin environmental component.” Environmental factors include metabolic diseases [18], immune disorders [19], infectious diseases [20], nutritional factors [21], GI microbiota [22], and a variety of toxic substances, including pesticides, heavy metals, and atmospheric pollutants [23]. Estimating the contribution of environmental factors to ASD insurgence is particularly complex since it is often difficult to discriminate one factor from the other or to identify the correct cause-effect relationship; for instance, disruption of the immune system or of hormonal homeostasis by pollutants may be erroneously categorized; GI microbiota may affect the presence and the diffusion of toxic metabolites [24]. One should also be aware that the contribution of environmental factors may be underestimated for temporal reasons; for instance, in order to affect neurodevelopment, the exposure to the environmental factor(s) should fall within a still undefined critical window of susceptibility and may thus be missed; evaluations performed in tissues or biological fluids having a rapid turnover may fail to display the presence of the toxic compound. In their recent review, Rossignol et al. [23], while pointing out a number of limitations and weaknesses found in the literature dealing with the effects of environmental factors on ASD etiology, nevertheless concluded that an association could be found between some pollutants and ASD (with stronger evidence for air pollutants and pesticides). Moreover, they reviewed a number of papers showing that ASD children bore a number of genetic polymorphisms that could decrease the expression of enzymes, such as PON1 and GST, able to efficiently eliminate environmental toxicants. These results add a new dimension to the toxicological studies on ASD. In fact, the decreased or impaired expression of an enzyme involved in detoxification may sum up with the increase of oxidative stress, be it of environmental or of genetic origin, and with other features, such as male-related hormonal factors which make males more susceptible to pollutants [25, 26] as well as to ASD. These considerations support the concept that the ASD trait is the result of a multiplicity of genetic and environmental factors.

## 3. ASD Biomarkers 

Generally speaking, biomarkers are biological parameters that differ between normal and pathological processes and can be used as indicators for diagnosis, prognosis, risk assessment of a disease, and evaluation of therapeutic outcomes. As briefly discussed above, the widely accessible chromosomal microarray analysis fails to identify genetic markers in about 70% of children carrying nonsyndromic ASD. Since the clinical phenotype of ASD overlaps, especially in the early ages, with many other clinical conditions, such as Attention Deficit and Hyperactivity Disorders (ADHD), Semantic Pragmatic Disorder, or severe Specific Language Impairment, the lack of specific biomarkers for ASD makes diagnosis very difficult to pediatricians, in particular when dealing with a very mild phenotype of the autistic spectrum.

ASD biomarkers are also needed for prognostic purposes. In fact, Autism Spectrum Disorders are generally considered lifelong conditions, but people with ASD exhibit outcomes that vary widely [27], especially when diagnosis and/or psychotherapeutic/pedagogic interventions are early, that is, at age 2 [28]. In effect, some cases may evolve in other psychiatric conditions, such as ADHD [29], while others may experience a very good outcome, since a minority of individuals with ASD may even lose the diagnosis [30, 31].

A few very good reviews have addressed the issue of ASD biomarkers in the past few years [32, 33]. In the present review we focused on studies dealing only with peripheral biomarkers. In fact, peripheral biomarkers are potentially easier and less expensive to analyze, when compared with genome-wide sequencing or brain imaging, which require procedures of data acquisition difficult to apply on a large scale. Moreover, some biological material, such as urine, is easy to obtain also in very young children. Another important feature of peripheral biomarkers is its potential for pointing to the biochemical pathways that, when altered, lead to the core ASD phenotype, which is shared also by syndromic ASD subjects and by ASD cases identified by CNV analysis.

## 4. ROC Curves

Our review is also characterized by the choice of examining only studies which included the calculation of the Receiver Operating Characteristic (ROC) curve. In our opinion, ROC curve should become the gold standard for the identification of parameters that are sensitive and specific enough to support ASD diagnosis, while its utility in prognosis, risk assessment, and evaluation of therapeutic interventions still awaits further studies.

ROC curves emphasize the most significant statistical differences between cases and controls. The Area Under the Curve (AUC) provides a useful metric to compare different biomarkers. While the AUC value close to 1 indicates an excellent predictive marker, a curve that lies close to the diagonal (AUC = 0.5) has no diagnostic utility. AUC value close to 1.00 is always accompanied by satisfactory values of specificity and sensitivity of the biomarker [34]. For a discussion of the use of ROC curves in translating biomarkers to clinical practice, see [35]. When studying the perspective ASD biomarkers, high sensitivity means that autism will be identified in most cases, while high specificity means that few, if any, healthy individuals will be positive to the test. Very interestingly, the combined ROC analysis of two distinct parameters increased their specificity (see, e.g., [36]), which suggests that one might resort to the combination of a panel of (related) parameters rather than to a single parameter alone.

Typically, when a diagnostic model is built upon a set of perspective markers, elaborated by using a “training” subset of data, a common practice to estimate its performance consists in feeding the model with randomly selected data (“testing” subset) and examining its ability to correctly classify these data as belonging to either of the two groups (e.g., healthy and pathological). Such procedure has the disadvantage of requiring large sets of data, since about one-third is set apart for validation. In the light of the fact that ROC analysis is able to predict the sensitivity and the specificity of the markers, we advance here the proposal to evaluate whether it might make the cross-validation procedure useless.

## 5. Search Strategy, Selection Criteria, and Limitations of Reviewed Studies

Identification of the studies was carried out through an extensive literature search using the PubMed database (National Library of Medicine, National Institutes of Health, Bethesda, MD, USA; http://www.ncbi.nlm.nih.gov/pubmed) mainly based on specific keywords and was updated to June 29, 2015. The search strategy included the terms Autism (mesh) AND Receiver Operating Characteristic Curve (mesh) OR ROC (mesh). Only articles reporting peripheral parameters were taken into consideration. Articles that did not present unique or new data were excluded from the analysis.

One hundred and thirteen citations were obtained and manually reviewed; 20 [36–55] fulfilled the selection criteria and were used to collect the data about ROC curve analysis.

Despite the large amount of studies about ASD, relatively few studies calculated ROC curves and, consequently, few data are available about sensitivity and specificity of a parameter. Most of the studies reporting ROC analysis dealt with people in paediatric age, and relatively few data are available about adolescents and adults patients. Most of them come from few research groups, and the data are thus limited to few geographical areas. Moreover, many of the reviewed studies had small sample sizes. In most of the studies the control group consisted in healthy, neurologically normal, children. Studies are needed that compare also subjects with clinical conditions that overlap in part to that of ASD (such as ADHD, as mentioned above), subjects with known aetiology such as Down's syndrome, and subjects with cognitive impairment without autistic features. Independent, larger, geographically different studies, extended to other early-onset neurodevelopmental disorders, are thus required to confirm (or disconfirm) available data.

## 6. Results and Discussion

Tables 1 [Table tab2] [Table tab3] [Table tab4] [Table tab5]–6 report published studies presenting peripheral parameters where ROC curves were calculated. Putative biomarkers are grouped into six different biochemical categories: neurotransmitters and neurotrophins, oxidative stress markers, fatty acids and phospholipids, inflammation markers, metabolites, toxic biomarkers, and metals and cations. Selection is updated at June 29, 2015.

Most parameters reported in Tables 1–6 have ROC curves that identify them as highly sensitive and highly specific putative markers, fulfilling the requirements reported in [34] and suggesting that they may be considered for further evaluation as bona fide ASD biomarkers.

Some data reported by the Saudi Arabia group [40, 44, 45] have AUC value of 1, which should correspond to 100% sensitivity and 100% specificity; however, they are puzzlingly reported with a specificity lower than 100%, a result that is not discussed.

**Figure 1:**
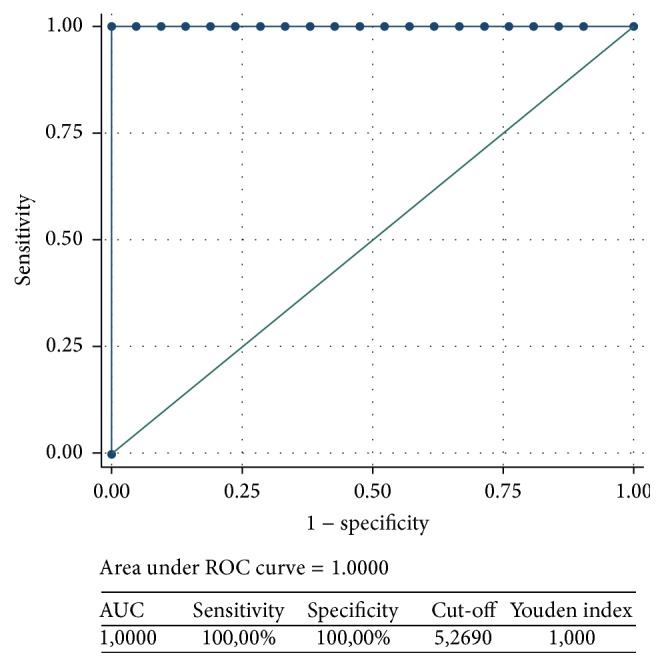
Receiver Operating Characteristic (ROC) curve showing sensitivity as a function of 1 − specificity of erythrocyte Na^+^K^+^-ATPase activity in ASD and typically developing children. This is an example of a ROC curve obtained when the values of the two groups (autistic and typically developing children) do not overlap. When the AUC value is 1.00, the curve degenerates into a segment which lies parallel to the *x*-axis on top of the graph. The parameter of the figure was previously published by our group [56]. Values are shown in Table 7. ROC curve analysis was based on nonparametric methods. The confidence intervals of ROC curves were set at 95%.

In the light of the usefulness of the ROC curves in evaluating the quality of putative biomarkers, we reexamined some of the data previously published by our group [56]. Notably, the best value (AUC = 1) was reached by a parameter (erythrocyte Na^+^, K^+^-ATPase activity), where the values of autistic and typically developing children did not show any overlapping (Figure 1).

**Figure 2:**
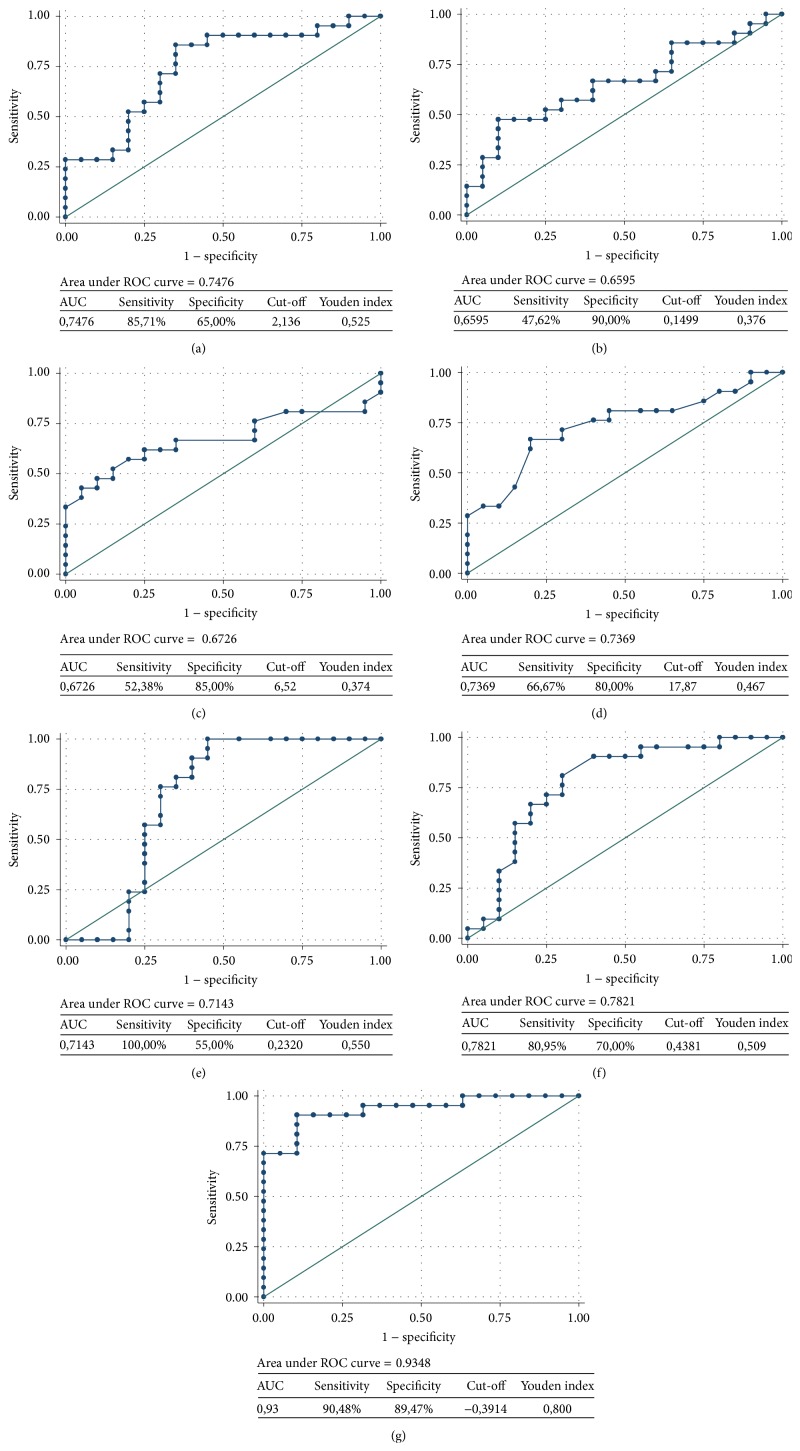
Receiver Operating Characteristic (ROC) curves showing sensitivity as a function of specificity in ASD and typically developing (control) children. (a) Urinary 8-isoprostane, (b) urinary hexanoyl-lysine adduct; (c) erythrocyte membrane omega 6/omega 3; (d) total monounsaturated fatty acids of the erythrocyte membrane; (e) fluidity of erythrocyte membrane (inner leaflet); (f) thiobarbituric acid reactive substances in erythrocyte membranes; and (g) combined ROC curve of the six parameters. Some parameter values* increase* in autistic children with respect to typically developing ones, while others* decrease*. ROC curve analysis of a combination of multiple parameters, albeit with opposite sign, increases both sensitivity and specificity. Values of these parameters, reported in [56], are shown in Table 7. ROC curve analyses were based on nonparametric methods. The confidence intervals of ROC curves were set at 95%.

Other six parameters which differed in a significant way between the two groups of children had fair-to-good ROC curve values (Figures 2(a)–2(f)). The combination of the six distinct parameters in one ROC curve analysis is shown in Figure 2(g). In order to be able to combine the six sets of data, raw data were standardized according to the following formula [57]:

(1) $$\begin{array}{c} z=\frac{x-\mu }{\sigma }, \end{array}$$

where *x* is the raw score, *z* is the standard score, *μ* is the mean of the population, and *σ* is the standard deviation of the population. The absolute value of *z* represents the distance between the raw score and the population mean in units of the standard deviation. *z* is negative when the raw score is below the mean and positive when above.

ROC analysis shows that the combination of different putative biomarkers increases both their sensitivity and their specificity as diagnostic tools. Notably, ROC analysis of most markers reported in Tables 1–7 shows that they are more sensitive than specific. Although sensitivity is a desirable quality for biomarkers (sensitive biomarkers do not erroneously classify positive cases), more specific biomarkers are needed for a correct classification of cases.  Figure 2 shows, as a representative example, how both sensitivity and specificity may be dramatically increased when* more than two* parameters are combined in one AUC curve; in fact, with such combination, AUC scores reach a value 0.93.

The choice of examining only studies where ROC analysis was carried out has greatly limited the number of reported parameters. Notably, however, they fall in categories, which bear many similarities to the theoretical classification adopted by Ratajczak [32]. In fact, even this limited number of peripheral biomarkers seems to be representative of ASD-relevant pathophysiological pathways that are presumably shared by all ASD patients.

The high or excellent AUC score obtained by the parameters reported in Tables 1–7 and in Figure 1 is not sufficient, however, to promote such putative biomarkers to bona fide ASD biomarkers. In fact, we already stressed the limitations of the studies here examined, which need to be confirmed by independent studies using larger population samples.

In our opinion, the use of combined ROC curves, rather than being an artefactual expedient, has the merit to highlight the fact that, in heterogeneous and multifactorial conditions as ASD are, only a (correct) combination of peripheral parameters may be able to maximize the predictive value of the tests. Moreover, in order to be useful for diagnosis and prognosis, putative biomarkers should be evaluated in studies assessing patients with confounding or overlapping clinical features and in longitudinal studies.

## 7. Conclusions

ASD are a group of early-onset neurodevelopmental diseases, whose causes are still poorly understood; growing evidences suggest that autism is a multifactorial disease influenced by genetic and environmental factors.

To date, autism diagnosis is based exclusively on clinical observation of altered behavior and can be made only around two years of age, since in younger children clinical diagnosis is difficult and uncertain. Therefore, valid biomarkers are needed that would allow improving and anticipating diagnosis. In addition, good biomarkers could provide predictive information on the clinical outcome of autism and help monitor the outcome of pharmaceutical or nutraceutical treatments.

The importance of the availability of strong biomarkers in ASD research cannot be underestimated. In effect, even the discovery of the biological networks underlying ASD pathophysiology could be boosted by their identification, as well as the development of new and personalized treatments able to cure or, at least, alleviate the symptoms of the disease.

This review analyzes the literature data to identify a panel of peripheral markers associated with ASD, by focusing on studies which made use of ROC analysis, a way to evaluate in an optimal way both sensitivity and specificity of a putative marker. At present, however, ROC analysis has not been used extensively enough to provide an exhaustive analysis of ASD biomarkers.

It is suggested here that ROC analysis be adopted as the gold standard to assess the quality of putative biomarkers, thus providing invaluable benefits to ASD research and its clinical applications.

## Figures and Tables

**Table 1:** Neurotransmitters and neurotrophins. AUC: Area Under the Curve; ASD: Autistic Spectrum Disorder; HC: healthy controls; and SD: standard deviation.

Lab test	Increase	Decrease	*P* values	Receiver Operating Characteristic (ROC)				Diagnosis	Age (Y): range (mean ± SD)	Number M/F	Control group	Country	Biologic sample	Technique	Reference
				Cut-off values	Sensitivity	Specificity	AUC								
GABA (*μ*mol/L)	*∗*		0.001	0.170	90.0%	80.0%	0.883	ASD	3–12 (7.0 ± 2.34)	52 male	HC	Saudi Arabia	Plasma platelet-free	ELISA	[37]
Serotonin (ng/mL)		*∗*	0.001	59.925	100.0%	100.0%	1.000								
Dopamine (ng/L)		*∗*	0.001	477.771	92.9%	88.9%	0.968								
Oxytocin (*μ*LU/mL)		*∗*	0.001	92.105	90.0%	100.0%	0.981								

Serotonin (ng/mL)	*∗*		<0.001	111.20	78.79%	80.65%	0.86	Autism	(12.21 ± 2.67)	27/6	HC	China	Whole blood	HPLC	[36]

Brain-derived neurotrophic factor (BDNF) (ng/mL)	*∗*		<0.0001	15.0	71.7%	86.7%	0.830	ASD	(3.78 ± 1.22)	48/12	HC	China	Serum	ELISA	[38]

Brain-derived neurotrophic factor (BDNF) (ng/mL)	*∗*		<0.0001	12.65	80.8%	70.2%	0.840	ASD	(4.0 ± 1.25)	62/13	HC	China	Serum	ELISA	[39]

Gamma aminobutyric acid (GABA) (ng/mL)	*∗*		<0.001												
Serotonin (5HT) (ng/mL)	*∗*		<0.001	High level of sensitivity and specificity				Autism	4–12	25	HC	Saudi Arabia	Plasma platelet-rich	ELISA	[54]
Dopamine (DA) (ng/mL)	*∗*		<0.001												

**Table 2 tab2:** Oxidative stress markers. AUC: Area Under the Curve; ASD: Autistic Spectrum Disorder; HC: healthy controls; and SD: standard deviation.

Lab test	Increase	Decrease	*P* values	Receiver Operating Characteristic (ROC)				Diagnosis	Age (Y): range (mean ± SD)	Number M/F	Control group	Country	Biologic sample	Technique	Reference
				Cut-off values	Sensitivity	Specificity	AUC								
GSH/GSSG (*μ*mol/L)		*∗*	0.001	31.100	100.0%	20.0%	1.000	Autism	3–15	20 male	HC	Saudi Arabia	Plasma	Biochemical assay	[40]
Peroxiredoxin 1 (ng/mL)	*∗*		0.001	26.240	90.0%	80.0%	0.915							ELISA	
Peroxiredoxin 3 (ng/mL)	*∗*		0.001	26.990	100.0%	85.0%	1.000							ELISA	
Thioredoxin 1 (ng/mL)	*∗*		0.001	52.140	100.0%	85.0%	0.993							ELISA	
Total glutathione (*μ*mol/L)		*∗*	0.001	9.320	100.0%	20.0%	1.000							Biochemical assay	
Thioredoxin reductase activity (mU/mL)	*∗*		0.001	2.350	65%	85%	0.881							Biochemical assay	

Thioredoxin (TRX) (ng/mL)	*∗*		<0.0001	10.6	86.9%	78.6%	0.913	ASD	(3.82 ± 1.34)	63/17	HC	China	Serum	ELISA	[41]

Isoprostane (pg/mL)	*∗*		0.001	78.270	100.0%	78.9%	1.000	Autism	4–12	20 male	HC	Saudi Arabia	Plasma	ELISA	[45]

**Table 3 tab3:** Fatty acids and phospholipids. AUC: Area Under the Curve; ASD: Autistic Spectrum Disorder; HC: healthy controls; SD: standard deviation; AA: arachidonic acid; DHA: docosahexaenoic acid; EPA: eicosapentaenoic acid.

Lab test	Increase	Decrease	*P* values	Receiver Operating Characteristic (ROC)				Diagnosis	Age (Y): range (mean ± SD)	Number M/F	Control group	Country	Biologic sample	Technique	Reference
				Cut-off values	Sensitivity	Specificity	AUC								
AA/DHA		*∗*	<0.001	0.66	100.0%	84.6%	0.977							Gas chromatography	
(EPA)/AA	*∗*		<0.001	1.07	93.8%	84.6%	0.937							Gas chromatography	
Phosphatidyl-serine (mmol/L)		*∗*	<0.001	0.068	100.0%	93.7%	0.998							HPLC	
Phosphatidylcholine (mmol/L)		*∗*	<0.001	1.619	80.0%	75.0%	0.825							HPLC	
Linoleic acid/AA	*∗*		0.03	0.69	60.0%	84.6%	0.592	Autism	4–12	25	HC	Saudi Arabia	Plasma	Gas chromatography	[42]
Alpha-linolenic acid/DHA	*∗*		0.004	0.57	90.9%	61.5%	0.713							Gas chromatography	
EPA/DHA	=	=	ns	0.39	100.0%	46.2%	0.658							Gas chromatography	
Phosphatidylethanolamine (mmol/L)		*∗*	0.002	0.037	80.0%	75.0%	0.806							HPLC	

Valeric acid (mmol/L)	*∗*		<0.001	0.196	100%	100%	1.000								
Acetic acid	*∗*		<0.001	0.684	92.3%	92.3%	0.985								
Lauric acid		*∗*	<0.001	1.214	100%	100%	1.000	Autism	4–12	26	HC	Saudi Arabia ASD	Plasma	Gas chromatography	[43]
Arachidonic acid		*∗*	<0.001	0.271	100%	100%	1.000								
Oleic acid		*∗*	<0.001	0.463	100%	100%	1.000								

**Table 4 tab4:** Inflammation markers. AUC: Area Under the Curve; ASD: Autistic Spectrum Disorder; HC: healthy controls; SD: standard deviation; and na: data not available.

Lab test	Increase	Decrease	*P* values	Receiver Operating Characteristic (ROC)				Diagnosis	Age (Y): range (mean ± SD)	Number M/F	Control group	Country	Biologic sample	Technique	Reference
				Cut-off values	Sensitivity	Specificity	AUC								
Interleukin-6 (pg/mL)	*∗*		*P* < 0.001	103.98	87.88%	74.19%	0.85	Autism	(12.21 ± 2.67)	27/6	HC	China	Plasma	ELISA	[36]
Combination of serotonin and interleukin-6				250.4	84.85%	96.77%	0.96								

HSP-70 (ng/mL)	*∗*		0.001	12.218	95.0%	84.2%	0.987	ASD	3–16	20 male	HC	Saudi Arabia	Plasma	ELISA	[44]
TGF-*β* (pg/mL)	*∗*		0.001	78.649	100.0%	89.5%	1.000								
Caspase-7 (ng/mL)	*∗*		0.001	6.698	90.0%	89.5%	0.968								
INF-*γ* (ng/mL)	*∗*		0.001	56.558	100.0%	78.9%	1.000								

Leukotriene (pg/mL)	*∗*		0.001	205.689	100.0%	84.2%	1.000	Autism	4–12	20 male	HC	Saudi Arabia	Plasma	ELISA	[45]
PGE2 (pg/mL)	*∗*		0.001	247.968	100.0%	84.2%	1.000								

Caspase-3 (ng/mL)		*∗*	<0.001	161.17	100.0%	86.7%	0.968	ASD	4–12	25 male	HC	Saudi Arabia	Plasma	na	[46]
IL-6 (pg/mL)		*∗*	<0.001	301.95	84.0%	100.0%	0.952								
TNFa (pg/mL)		*∗*	<0.001	297.67	76.0%	100.0%	0.915								

Neopterin (nmol/L)	*∗*		<0.0001	≥8.5	≥84.2%	80.1%	0.876	ASD	(3.69 ± 1.30)	64/16	HC	China	Plasma	ELISA	[47]

Lipoxin A4 (pg/mL)		*∗*	<0.0001	81.5	90.7%	76.0%	0.911	Autistic disorders	(3.69 ± 1.22)	59/16	HC	China	Plasma	ELISA	[48]

Interferon-*γ*-induced protein-16 (ng/mL)	*∗*		0.001	1.750	100.0%	70.0%	0.933	ASD	3–12 (7.0 ± 2.34)	52 male	HC	Saudi Arabia	Plasma	ELISA	[37]

**Table 5 tab5:** Metabolites. AUC: Area Under the Curve; ASD: Autistic Spectrum Disorder; HC: healthy controls; SD: standard deviation; na: data not available; and CI: confidence interval.

Lab test	Increase	Decrease	*P* values	Receiver Operating Characteristic (ROC)				Diagnosis	Age (Y): range (mean ± SD)	Number M/F	Control group	Country	Biologic sample	Technique	Reference
				Cut-off values	Sensitivity	Specificity	AUC								
Pentacarboxyl (penta-) porphyrins (nmol/gCr)	*∗*		<0.006	2.1	30% for AUT 36% for PDD-NOS	94%		Autism and PDD-NOS	2.5–12.4 (6.64 ± 2.59)	30 male with AUT 14 male with PDD-NOS	HC	USA (Washington, Oregon)	Urine	HPLC and spectrofluorometry	[49]
Coproporphyrins (nmol/gCr)	*∗*		<0.006	108	33% for AUT 14% for PDD-NOS	94%									
The combined *Z* score measure (*Z*-penta + *Z*-copro)				1.13	33% for AUT 21% for PDD-NOS	100%									

Methoxyphenyl oxime (volatile organic compounds)		*∗*	<0.0001	na	na	na	0.76–0.99 95% CI	Autism	(6.9 ± 2.1)	22/2	HC	Italy	Urine	Gas chromatography-mass spectrometry	[50]

Succinate orthogonal partial least-squares discriminant analysis (OPLS-DA) scores	*∗*		<0.001		100%	100%	0.92	Autism	6–14 (mean: 8)	24/6	HC	France	Urine	1H-NMR spectroscopy 1H-13C heteronuclear single quantum coherence (HSQC) NMR	[51]
Glutamate		*∗*	<0.05												
3-Methyl-histidine		*∗*	<0.05												

**Table 6:** Toxic biomarkers, metals and cations. AUC: Area Under the Curve; ASD: Autistic Spectrum Disorders; HC: healthy controls; and SD: standard deviation.

Lab test	Increase	Decrease	*P* values	Receiver Operating Characteristic (ROC)				Diagnosis	Age (Y): range (mean ± SD)	Number M/F	Control group	Country	Biologic sample	Technique	Reference
				Cut-off values	Sensitivity	Specificity	AUC								
Urinary phthalate (*µ*g/mL) 5-OH-MEHP	*∗*		<0.05	0.177	52.1%	75.6%	0.638								
Urinary phthalate (*µ*g/mL) 5-Oxo-MEHP	*∗*		<0.01	0.142	46.0	91.1	0.666	ASD	(11 ± 5)	36/12	HC	Italy	Urine	HPLC electrospray ionization MS	[52]
Urinary phthalate (*µ*g/mL) MEHP	*∗*		<0.05	0.01	79.2	44.2	0.631								
Urinary phthalate (*µ*g/mL) ALL	*∗*			0.724	39.2	97.8	0.671								

Zinc/copper (*μ*g/dL)		*∗*	<0.001	0.665	90.0%	91.7%	0.968	ASD	(3.78 ± 1.22)	48/12	HC	China	Serum		[53]

Lead (mmol/L)	*∗*		0.001	High level of sensitivity and specificity				Autism	4–12	25	HC	Saudi Arabia	RBC	HCL	[54]

Amyloid beta (1–40) (pg/mL)		*∗*	<0.05	165.00	82.7%	75.0%	0.773	Autism	3–16	52	HC	Saudi Arabia	Plasma	ELISA	[55]
Amyloid beta (1–42) (pg/mL)		*∗*	<0.05	60.29	82.4%	80.6%	0.905								

Ca2+ (mmol/L)		*∗*	<0.001	8.17	100.0%	100.0%	1.000								
Na+ (mmol/L)		*∗*	<0.05	124.50	100.0%	71.4%	0.786								
K+ (mmol/L)	*∗*		<0.001	7.00	84.0%	85.7%	0.900	ASD	4–12	25 male	HC	Saudi Arabia	Plasma		[46]
Ca2+/Mg2+		*∗*	<0.001	4.41	95.8%	100.0%	0.981								
Na+/K+		*∗*	<0.01	17.14	93.8%	78.6%	0.888								

**Table 7:** Features and ROC curve analyses of peripheral biomarkers reported in Figure 2 and published in [56]. Combined *Z* score has a *P* value < 0.0001, whereas each parameter has a *P* value < 0.01 or <0.05. Both sensitivity and specificity were strongly increased by the combination of ROC curves. AUC: Area Under the Curve; FA: fatty acids; TBARS: thiobarbituric acid reactive substances; and MDA: malondialdehyde.

Lab test	Increase	Decrease	*P* values	Receiver Operating Characteristic (ROC)					Diagnosis	Age (Y): range (mean ± SD)	Number M/F	Control group	Country	Biologic sample	Technique	Reference
				Cut-off values	Sensitivity	Specificity	AUC	*Z* score								
(1) Na+/K+-ATPase activity (*µ*gPi/mg prt/h)		*∗*	<0.0001	5.269	100%	100%	1.00							Red blood cells	Biochemical assay	
(2) 8-Isoprostane (ng/mg Cr)	*∗*		<0.01	2,136	85.71%	65%	0.7476							Urine	ELISA	
(3) Hexanoyl-lysine adduct (nmol/mg Cr)	*∗*		<0.05	0.1499	47.62%	90%	0.6595							Urine	ELISA	
(4) Total monounsaturated fatty acids (% of total FA)	*∗*		<0.01	17.87	66.67%	80%	0.7369		ASD	5–12 (6.8 ± 2.23)	17/4	HC	Italy	Red blood cells	Gas chromatography	[56]
(5) Omega 6/omega 3	*∗*		<0.05	6.52	52.38%	85	0.6726							Red blood cells	Gas chromatography	
(6) Inner membrane leaflet fluidity		*∗*	<0.05	0.232	100%	55%	0.7143							Red blood cells	Fluorescence anisotropy (reciprocal of fluidity) of TMA	
(7) TBARS (MDA, *µ*M)	*∗*		<0.01	0.4381	80.85%	70%	0.7821							Red blood cells	Biochemical assay	
Combined scores of lab tests 2–7			<0.0001	−0.3914	90.48%	89.47%	0.93	0.4799								
